# Development of the Tracheostomy Well-Being Score in critically ill patients

**DOI:** 10.1007/s00068-022-02120-9

**Published:** 2022-10-13

**Authors:** Christopher Ull, Christina Weckwerth, Uwe Hamsen, Oliver Jansen, Aileen Spieckermann, Thomas Armin Schildhauer, Robert Gaschler, Christian Waydhas

**Affiliations:** 1 Department of General and Trauma Surgery, BG University Hospital Bergmannsheil, Bürkle-de-la-Camp-Platz 1, 44789 Bochum (North Rhine-Westphalia), Germany; 2 Faculty of Psychology, FernUniversität of Hagen, Universitätsstraße 47, 58097 Hagen, Germany; 3 Medical Faculty University Duisburg-Essen, Hufelandstraße 55, 45147 Essen, Germany

**Keywords:** Critical illness, Intensive care unit, Score, Tracheostomy, Ventilation, Well-being

## Abstract

**Purpose:**

Little attention has been given to understanding the experiences and perceptions of tracheostomized patients. This study aimed to measure the impact of tracheostomy on well-being in critically ill patients with the development of the Tracheostomy Well-Being Score (TWBS).

**Methods:**

This is a prospective, monocentric, observational study including critically ill patients with a tracheostomy without delirium. A 25-item questionnaire with items from six categories (respiration, coughing, pain, speaking, swallowing, and comfort) was used to select the 12 best items (two per category) to form the TWBS score after testing on two consecutive days. Item selection secured (1) that there were no skewed response distributions, (2) high stability from day 1 to day 2, and (3) high prototypicality for the category in terms of item-total correlation.

**Results:**

A total of 63 patients with a mean age of 56 years were included. The 12 items of the TWBS were characterized by a high retest reliability (*τ* = 0.67–0.93) and acceptable internal consistency. The overlap with the clinician rating was low, suggesting that acquiring self-report data is strongly warranted.

**Conclusion:**

With the TWBS, an instrument is available for the assessment of the subjective effects a tracheostomy has on in critically ill patients. The score potentially offers a chance to increase well-being of these patients. Additionally, this score could also increase their quality of life by improving tracheostomy and weaning management.

**Clinical Trial Registration:**

German Clinical Trials Register Identifier DRKS00022073 (2020/06/02).

**Supplementary Information:**

The online version contains supplementary material available at 10.1007/s00068-022-02120-9.

## Introduction

On average, 5–10% of mechanically ventilated patients in intensive care units (ICUs) are tracheostomized [1, 2] and up to 34% of those require mechanical ventilation for more than 48 h [3]. Some studies report a median duration of ventilation in tracheostomized patients of 35–37 days with an upper range of more than 50 days [4, 5]. Tracheostomy corresponds to global routine practice in intensive care medicine because it potentially confers several benefits over prolonged endotracheal intubation in critically ill patients, including reduction of dead space, airway resistance and labored breathing, improved patient comfort, decreased need for sedation, effective communication, airway clearance with reduced risk of aspiration, and improved oral care [6–8].

Despite these medical advantages for tracheostomized intensive care unit patients during weaning, only little attention has been granted toward understanding the experiences and perceptions of these patients [9]. In previous studies, tracheostomized patients have frequently reported dyspnea, inadequate communication skills, persistent dysphagia and coughing, being fearful and experiencing pain during suctioning and tube change, and having physical discomfort when moving their head and neck during the ICU inpatient stay [10–15]. To address those issues, a versatile toolbox with tracheostomy tubes of different materials, dimensions, lengths and angulations, with or without inner cannulas as well as with or without fenestration, is available [16]. Tube selection may have an impact on important characteristics such as pressure and resistance to airflow [17]. However, only few guidelines exist regarding the selection of a particular type or size of the tracheostomy tube [18]. The choice of an optimal tracheostomy remains mostly a patient-specific empirical question that could benefit substantially from efficiently gaining information from the participant.

Although scoring systems for improving clinical management in tracheostomized patients are available and primarily focus on dysphonia, prolonged ventilator support, or decannulation, they do not pay attention to the best choice of tracheostomy tube and rarely measure the impact the tracheostomy has on the well-being of those particular patients [19–24]. To address this mismatch, we aimed to develop a tracheostomy-well-being score specifically for critically ill tracheostomized patients.

## Methods

### Study design and selection criteria

This prospective, monocentric, observational study was performed at three medical and surgical ICUs and one intermediate care unit (IMC) of the BG University Hospital Bergmannsheil Bochum, Germany. The study was approved by the authors institutional review board (20-6887) and registered at the German Clinical Trials Register (DRKS00022073). It adheres to the “Strengthening the Reporting of Observational Studies in Epidemiology (STROBE) statement” (Online Resource 1) [25].

From June 8, 2020, to April 8, 2021, all eligible patients were prospectively tested. All patients included met the following criteria: (1) inserted tracheostomy tube for more than 48 h and expected to remain cannulated at least for the next 7 days; (2) adequate cognitive and motoric skills to answer the survey manually, verbally, or with support of eye-tracking (ET) devices; (3) over 18 years of age; (4) a score of − 1, 0 or 1 points on the Richmond agitation-sedation scale (RASS) [26] or a score of < 3 points on the nursing delirium screening scale (Nu-DESC) [27, 28] in patients without sedation; and (5) successful completion of the survey on two consecutive days.

**Fig. 1:**
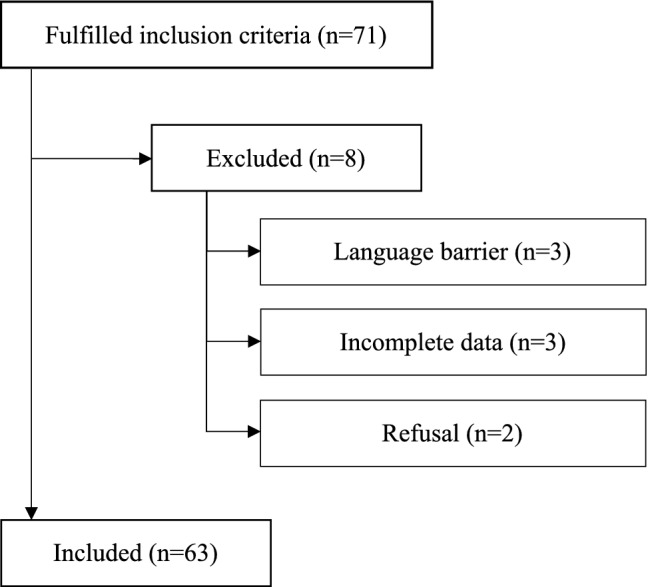
Flow diagram showing results of patient search (*n* = 63)

Out of 71 eligible tracheostomized patients, 8 patients were excluded because of the following conditions: language barrier (*n* = 3), incomplete data (*n* = 3), and refusal to participate (*n* = 2). The remaining 63 patients were enrolled in the study (Fig. 1).

### Demographic data

Standard parameters, such as sex, age, reason for hospital admission, and length of stay in hospital, were applied to describe the demographic data of the included patients. The reasons for ICU or IMC admission were divided into major trauma, non-abdominal sepsis, acute abdomen, exacerbation of chronic obstructive pulmonary disease, modification of long-term mechanical ventilation, and cancer.

### Development of the item-pool for the Tracheostomy Well-Being Score

We aimed to develop a questionnaire that would, on the one hand, provide a score and on the other hand allow charting of tracheostomy well-being across a profile of different categories.

The categories of TWBS were based in part on the review by Nakarada-Kordic et al. [9] The authors highlighted the typical problems of a tracheostomized patient, which primarily have a negative impact on patient well-being. Based on their results, six categories (*respiration, coughing, pain, speaking, swallowing, comfort*) could be defined. Corresponding questions were collected for each category.

The survey was developed by the interdisciplinary ICU team consisting of critical care nurses, intensivists, psychologists, speech and occupational therapists, and physiotherapists. The list of items was also based on comments and suggestions of tracheostomized ICU patients who were in the weaning process or were on long-term ventilation or already had been successfully weaned. From a list of 47 potential questions, 17 were removed because they were very similar and 5 items were removed because they appeared irrelevant in the intensive care setting from the authors’ point of view, so that 25 items remained from the categories. The answers to the 25 items should be graded on a 4-point Likert scale (0 = never, 1 = sometimes, 2 = often, 3 = always). The selection of the 25 questions was assessed positively by all involved, so that the final version of the questionnaire was created (Online Resource 2).

### Standardized assessment

Patients potentially fulfilling our inclusion criteria were identified during the daily ward round of the interdisciplinary ICU and IMC teams. Prior to inclusion and to each session, the patient’s level of consciousness was assessed using the RASS and the Nu-DESC. In each of the six categories of the survey, an external assessment of suspected problems was also performed by the first author of the study, a medical specialist for orthopedics and trauma surgery with many years of expertise in critical care medicine, using a 4-point Likert scale (0 = unlikely, 1 = moderate, 2 = likely, and 3 = quite likely). The procedure was explained verbally to the patients, and informed consent was obtained in written form, or if not possible, verbally, via head nodding, or by blinking. This was confirmed by persons who were unrelated to the investigation. Depending on the current fine motor capabilities of their arms and communication skills, the patients were either able to answer the 25-item survey manually with paper and pen or were able to answer verbally after the questions were read aloud by the experimental supervisor, or the patients were able to answer the questionnaire via ET devices. The Tobii Dynavox I-15 + ET device (Tobii Dynavox, Danderyd, Sweden) was used for this in nonverbally restricted patients with limited fine motor capabilities [29–31]. On the next day, the survey was repeated with each patient. Throughout the entire study, the investigation was performed by the first author of this study using this 25-item questionnaire (Online Resource 2).

### Item selection for the Tracheostomy Well-Being Score and reliability analysis

In order to determine which items of the pool should be selected for the scale, we checked the relevant criteria as detailed below. (1) For an initial overview, the percentage response frequencies were determined for all 25 items (Table 2). The items of the final scale should differentiate well among respondents. Therefore, items to which most respondents provide the same response should not be considered. Based on this reasoning, items with a skewed distribution of answers (e.g., > 50% answers at one of the extremes—never or always) were excluded.

(2) In a second step (Table 3), we assessed retest reliability by correlating for each of the 25 items responses from day one with responses from day two across patients. High retest-reliability implies that differences among participants reproduce from 1 day to the next, suggesting that the item is understood well and taps into judgments that are not strongly fluctuating. As items suitable for the scale should elicit reproducible responses, we wanted to make sure that retest reliability is high.

(3) In a third step, we considered content categories. As the items in the pool had been generated by taking six categories into account it was relevant to check to what extent each item would be prototypical for the category it belongs to. To this end, we computed for each item how strongly it correlated with the average of the other items of the category it belonged to (using day one data). Prototypical items need to show a positive item-total correlation. Beforehand, inversely worded items (S1, S2, SW3, CO3, and CO5) were recoded so that all items were coded in terms of criterion and thus positively correlated with each other. We aimed to select items that would show a high item-total correlation as these can be considered as prototypical for their category. Given that retest reliability was high for the most items, we focused on how well the item would represent its category. The wording of each chosen item was to fit to the category and represent aspects of only the one category. Furthermore, item-total correlation was to indicate that the item represents the category well.

(4) Aiming at items that would distinctively represent one category, we excluded items that mentioned aspects of two categories.

The four steps reported above were taken with the aim to form a short scale that would, on the one hand, provide a score and, on the other hand, allow to compare participants on a profile representing the six categories of tracheostomy well-being. Accordingly, we selected sequentially according to the four steps charted above the two best items per category.

After completing the item selection (i.e., selecting the 12 best items out of the pool of 25), we ran to further analyses to evaluate the result. First, we tested the internal consistency of the 12 items scale using Cronbach’s alpha. A positive and high value in Cronbach’s alpha (i.e., high internal consistency) shows that there is similarity within persons (i.e., those who answer “always” or “often” on some items also do so on others) while at the same time there are substantial differences between persons (i.e., some patients in most items answer “always” or “often” other patients respond to most items with never or sometimes).

Second, we checked separately for each of the six content categories whether the report of the patient (i.e., average across the answers to the two items per category) would overlap with the clinician rating.

A high correlation between the self-report of the patient and the clinician rating would suggest that these perspectives overlap strongly. This would bring up the question if it is actually warranted to take the time and effort to assess the patient’s perspective.

### Statistical analysis

Statistical analysis was performed using Microsoft^®^ Office Excel^®^ for Mac 2019 (Microsoft Corporation, Redmond, WA, USA) and IBM^®^ SPSS^®^ Statistics Version 28 2021 (IBM Corporation, Armonk, NY, USA). Demographic data are presented as mean and standard deviation or as absolute numbers and percentage. Retest reliability was assessed with the Kendall’s *τ* correlation coefficient. Item–total correlation was determined using the part-whole correction implemented in SPSS based on the Pearson correlation coefficient. The inter-item correlation between the two items selected for each of the six categories was assessed using Pearson correlation coefficient. As explained above, internal consistency of the scale was computed using Cronbach’s alpha.

## Results

### Demographic data

The demographic data of the 63 enrolled patients are listed in detail in Table 1. Four out of 63 patients (6.3%) were able to answer the 25-item survey manually. The majority of patients (84.1%) used their voices for scoring. In six patients (9.5%), ET devices were necessary for answering the questionnaire.

**Table 1:** Demographic data of enrolled patients (*n* = 63)

	Total
Female-to-male ratio	10 (15.9%): 53 (84.1%)
Age (years)	56 ± 17.9
Reason for hospital admission	
Major trauma	30 (47.6%)
Injury Severity Score (points)	30.5 ± 6.7
Non-abdominal Sepsis	9 (14.3%)
Acute abdomen	8 (12.7%)
Chronic obstructive pulmonary disease	7 (11.1%)
Modification of mechanical ventilation	5 (7.9%)
Cancer	4 (4.6%)
Technique of tracheostomy	
Surgical	17 (27%)
Percutaneous dilatational	46 (73%)
Tracheostomy	
Acute	51 (80.9%)
Permanent	12 (19.1%)
Time from tracheostomy to testing (days)	642.8 ± 1797.2
Time from ICU/IMC admission to testing (days)	26.6 ± 38.6
Ventilation days on the ICU/IMC	31.1 ± 40.9
LOS on the ICU/IMC	47.4 ± 44.6
Decannulation	28 (44.4%)
Time from tracheostomy to decannulation	56.9 ± 52.9
Total ventilation days	46 ± 61.5
LOS hospital (days)	123.5 ± 112.8
Survival	59 (92.1%)

Data presented as absolute numbers (percentage) or mean ± standard deviation

Abbreviations: *ICU* intensive care unit, *IMC* intermediate care unit, *LOS* length of stay

On discharge or transfer to further rehabilitation, 28 patients (44.4%) were successfully decannulated, 26 patients (41.2%) were on long-term mechanical ventilation, and 4 patients (6.3%) were cannulated but not mechanically ventilated (e.g., cough assistance techniques, reduction of labored breathing and suctioning). During the inpatient stay, five patients (7.9%) died due to their critical illness.

### Item selection for the Tracheostomy Well-Being Score and reliability analysis

An overview of all 25 items used with the respective response options for day one and day two is shown in Table 2. In Step 1 (see above), we detected that 12 items show a skewed distribution of responses and should therefore be excluded. In Step 2, we learned that the retest reliability is high for all items. All proved reliable with values ranging from 0.67 to 0.93 using Kendall’s τ (Table 3). Hence, no item had to be excluded due to a low correlation of day 1 responses with day 2 responses.

**Table 2:** Percentage response frequencies for all 25 items of the questionnaire

Category	Response options day one				Response options day two			
	Never	Sometimes	Often	Always	Never	Sometimes	Often	Always
*Respiration*								
R1	20.6	33.3	36.5	9.5	20.6	41.2	30.1	7.9
R2	12.7	52.3	26.9	7.9	19	44.4	28.5	7.9
R3	31.7	34.9	22.2	11.1	30.1	38.1	23.8	7.9
*Coughing*								
C1	11.1	44.4	38.1	34.9	7.9	53.9	34.9	3.1
C2	28.5	41.2	26.9	3.1	28.5	44.4	23.8	3.1
C3	12.7	41.2	39.6	6.3	11.1	46	34.9	7.9
C4	1.6	46	52.3	0	3.1	46	50.7	0
*Pain*								
P1	34.9	36.5	20.6	7.9	31.7	38.1	20.6	9.5
**P2**	**50.8**	**25.4**	**20.6**	**3.1**	**49.2**	**30.1**	**14.3**	**6.3**
P3:	26.9	38.1	30.1	4.7	25.4	39.6	26.9	7.9
P4	23.8	33.3	28.5	14.3	17.4	41.2	25.4	15.8
**P5**	**58.7**	**14.3**	**15.8**	**11.1**	**55.5**	**22.2**	**11.1**	**11.1**
*Speaking*								
S1	39.6	36.5	19	4.7	39.6	33.3	22.2	4.7
S2	25.4	23.8	20.6	30.1	23.8	25.4	19	31.7
**S3**	**68.2**	**22.2**	**6.3**	**3.1**	**69.8**	**22.2**	**6.3**	**1.6**
S4	23.8	26.9	41.2	7.9	20.6	26.9	46	6.3
*Swallowing*								
SW1	26.9	46	23.8	3.1	26	41.2	26.9	4.7
SW2	42.8	42.8	12.7	1.6	42.8	44.4	11.1	1.6
SW3	14.3	26.5	39.6	9.5	17.4	30.1	44.4	7.9
SW4	30.1	30.1	31.7	7.9	34.9	33.3	26.9	4.7
*Comfort*								
**CO1**	**57.1**	**31.7**	**6.3**	**4.7**	**63.5**	**28.5**	**4.7**	**3.1**
**CO2**	**57.1**	**31.7**	**9.5**	**1.6**	**53.9**	**36.5**	**7.9**	**1.6**
CO3	6.3	33.3	42.8	17.4	9.5	25.4	52.3	12.7
**CO4**	**7.9**	**3.1**	**23.8**	**65**	**6.3**	**6.3**	**23.8**	**63.5**
CO5	4.7	23.8	61.9	9.5	3.1	25.4	65	6.3

Items in bold excluded due to skewed response frequency

**Table 3:** Selection criteria and values for the reliability analysis of the 25 items

Category	Item	*N*	Retest Reliability τ	Item-total correlation *r*	Correlation among two selected items *r*
*Respiration*					
**R1**	**How often is breathing complicated by your tracheostomy tube?**	**63**	**0.76**	**0.51**	**0.63**
**R2**	**How often do you experience shortness of breath?**	**63**	**0.77**	**0.62**	
R3	How often do you feel that your trachea is dry?*	63	0.77	0.33	
*Coughing*					
**C1**	**How often do you cough?**	**63**	**0.83**	**0.49**	**0.25**
C2	Do you have coughing attacks while speaking?	61	0.80	0.57	
**C3**	**How often do you feel that you cannot cough up mucus properly?**	**63**	**0.71**	**0.29**	
C4	How often do you need suctioning?*	63	0.84	0.43	
P*ain*					
P1	How often do you have a foreign body sensation?*	63	0.78	0.58	
P2	How often does your cannula hurt when you swallow?	63	0.80	0.60	
**P3**	**How often does your cannula hurt when you move or are moved?**	**63**	**0.82**	**0.50**	**0.27**
**P4**	**How often do you have pain when suctioning?**	**63**	**0.93**	**0.52**	
P5	How often do you have pain when changing your cannula?	39	0.86	0.02	
*Speaking*					
**S1**	**How often do you think you are well understood with whispered speech? *[R]***	**61**	**0.67**	**0.28**	**0.31**
S2	How often do you feel the volume of your speech is normal? ***[R]***	61	0.91	0.16	
S3	How often does your cannula hurt when you speak?*	61	0.76	0.32	
**S4**	**How often do you feel out of breath when speaking?**	**61**	**0.70**	**0.44**	
S*wallowing*					
**SW1**	**How often does your tracheostomy tube interfere with swallowing liquids or food?**	**62**	**0.93**	**0.49**	**0.43**
SW2	How often do you feel saliva flowing into the trachea?	63	0.84	0.50	
**SW3**	**How often are you satisfied with your currently inserted tracheal cannula? [R]**	**63**	**0.71**	**0.20**	
SW4	How often are you afraid of choking or coughing while eating or drinking?	62	0.83	0.41	
*Comfort*					
CO1	How often do you feel shame or disgust because of your tracheostomy tube?*	63	0.88	0.41	
CO2	How often are you afraid of your cannula slipping out?	63	0.84	0.29	
**CO3**	**How often do you feel that your tracheostomy tube fits well? *[R]***	**63**	**0.89**	**0.16**	**0.44**
CO4	How often does an unblocked tracheostomy tube feel more comfortable than a blocked tracheostomy tube? [R]	62	0.76	0.16	
**CO5**	**How often do you find the fixation of your tracheostomy tube comfortable? [R]**	**63**	**0.90**	**0.58**	

Final selected items in bold letters. [R] = recoded items. Items marked with „* “ were excluded as they overlap with two categories and hence lack distinctiveness

In Step 3, we found out that there are at least two items per category that can be regarded as prototypical for their category due to a high item-total correlation (see Table 3).

Scrutinizing whether items mention aspects of two categories rather than distinctly represent one category led to excluding five items in Step 4. For each of the two best items per category (printed in bold in Table 3), there was a positive inter-correlation. A positive correlation among the two items per category signals internal consistency and suggests that their average can be used to represent the category when analyzing profiles across categories.

Furthermore, Cronbach’s alpha calculated across all 12 items of the TWBS assured that there was decent internal consistency on scale level (0.74). Given that patients responded on a 4-point scale (0 = never, 1 = sometimes, 2 = often, 3 = always), the highest possible sum score across the 12 items is 36 (all items responded to with “always” while a score of zero would indicate that a patient had responded “never” to all items. In our sample, the mean was 16.6 and the standard deviation was 5.5. The minimum score obtained by a patient was 3, and the maximum was 30.

**Fig. 2:**
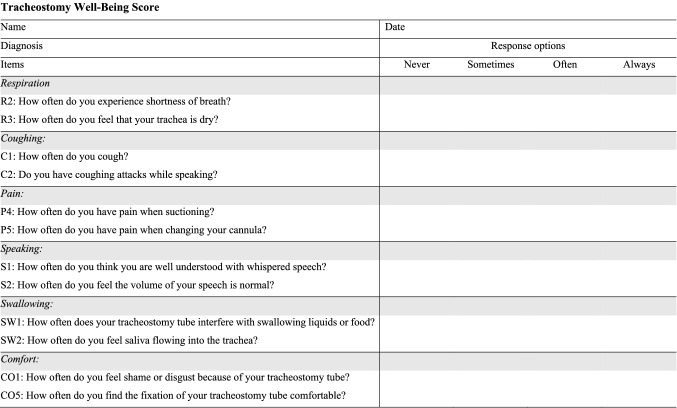
Final version of the Tracheostomy Well-Being Score (Scoring: 0 = never, 1 = sometimes, 2 = often, 3 = always)

Finally, checking the correlations between the self-reports and the clinician rating (Table 4) showed that there was little overlap among the patients’ and clinician perspective (no significant correlations). An overview of the final version of the 12-item TWBS survey as it will be applied to the patients in future work listed in Fig. 2.

**Table 4:** Correlation values for the ratings between self-reports and clinician rating of the respective category

*Category*	Day one		Day two	
	*r*	*p*	*r*	*p*
Respiration:	0.14	0.288	0.19	0.134
Coughing:	− 0.04	0.774	0.05	0.709
Pain:	− 0.18	0.162	0.03	0.851
Speaking:	− 0.07	0.563	0.01	0.966
Swallow:	0.07	0.569	0.12	0.372
Comfort:	0.09	0.478	− 0.05	0.679

*r* = Pearson correlations between the self-reports and the clinician rating

## Discussion

Based on the current research, we can offer an instrument to assess tracheostomy well-being. The scale has been developed in a cohort of critically ill patients with tracheostomy. Item selection was based on an interdisciplinary elaborated 25-item pool divided into six categories. The included 12 items of the TWBS showed an acceptable internal consistency and satisfying retest-reliability.

With these results, we can offer a concise tool for assessment of tracheostomized patients to improve tracheostomy management, weaning and long-term mechanical ventilation, resulting in a better professional support during the ICU stay and an increasing quality of life.

The primary goal of tracheostomy management is successful weaning and, alternatively, long-term ventilation via tracheostoma adapted to the individual patient in the event of an unsuccessful weaning process [6–8]. Until now, the optimal and individual fitting of a tracheal cannula was based on objective radiological parameters, studies on a model or computer simulations [17, 18, 32–34]. In the period of tracheostoma care, standardized pathways appear to be quite helpful, but no subjective information from the patient regarding well-being is provided [35–37]. Currently, no standardized survey instruments exist to assess well-being in a tracheostomized patient, although studies show that tracheostomy care has a significant negative impact on patient well-being [9, 37–39].

Against this background, the TWBS now provides a standardized questionnaire that can be used to assess the subjective satisfaction of critically ill patients with an inserted tracheostoma. During the period of tracheal cannulation, the patient now has the opportunity to provide active feedback and improvement approaches for tracheostoma management by means of the score. By actively involving the patient in the therapy decision-making process, it can be assumed that patient autonomy is increased and the patient’s quality of life is improved, since problems with the tracheal cannula can be routinely recorded individually and optimized in a targeted manner.

The TWBS covers the relevant aspects of subjective well-being in tracheostomized patients by using a minimum number of items and by using simple and clear language in both the question and response options. All items of the TWBS capture significant problems of tracheostomy management that are relatively easy to take care of with little effort (e.g., analgesia before suctioning). The TWBS complements the main elements, which are assessed as part of the patient’s daily ICU care routine, and is not dependent on clinical parameters (e.g., weaning parameters, laboratory values, and invasive vitality parameters) or additional diagnostics. The score is generally applicable, easy to use for every member of the medical ICU team and allows testing every day without any special training or extensive time commitment.

For the medical ICU team, the use of the TWBS is presumably helpful in efficiently designing an individualized, optimal tracheostoma management without having to make use of further diagnostics. Since subjective information from affected patients has not yet been taken into account, this score can be particularly valuable in the decision-making process for the optimal cannula supply through regular evaluation of the comfort of the inserted tracheal cannula [17, 18, 32–34]. The active involvement of patients in the therapy decision, by systematically identifying potential problems with the inserted tracheostomy tube, is absolutely necessary to optimize individualized tracheostomy management.

Asking participants to report on tracheostomy-well-being takes time and effort. If the patients’ perspective could be inferred to a high extent from the rating of a clinician, one might debate if the effort of collecting self-reports is warranted. Accordingly, we checked to what extent self-report and clinician rating overlapped. Table 4 shows that there are small-to-moderate correlations for most categories. Importantly, comfort from the patients’ perspective did not correlate with comfort from an observers’ perspective. Hence, the results suggest that an observer can only capture a minor proportion of the variance in participants’ tracheostomy well-being underlining that collecting self-report data are warranted. In addition, there are differences in the subjective assessment of comfort and well-being with an inserted tracheostomy tube, so that there is a clear potential for improvement.

Of course, the TWBS does not replace the previous measures for finding an optimal tracheostomy tube fitting, but can be seen as a supplementary tool in this framework, which maps the subjective part as a decision-making aid for a patient-oriented, best possible tracheostomy management. The TWBS can help counter the lack of studies dealing with the use of patient reports in tracheostoma management. We see an enormous need for research, especially to systematically assess the influence of a tracheostoma on the general health of affected patients and to identify direct potential for improvement.

## Limitations

Countering possible influences of a selection bias on the results, the data were collected prospectively from all patients who were admitted to the ICU/IMC and tracheostomized. Yet, the present study was conducted at a single institution. Multicentric studies could further investigate usability and impact of the TWBS. Most importantly, as the current work yielded a manageable scale to assess tracheostoma-well-being, future studies can test whether the use of the TWBS actually leads to an improvement in the patient’s well-being.

It is of highest practical relevance to assess how frequent a specific problem with the tracheostoma occurs. While the TWBS fits the aim to quickly assess which problems occur, future work could conditionally include intensity ratings. This might be particularly useful for the pain category. If the patient reports that a specific tracheostoma problem frequently occurs, an item tapping pain intensity could be flexibly added. In addition, consideration could be given to offering patients a free response option in addition to the dichotomous response option. In this way, tracheostoma management could be adapted even more individually. For patients with limited motor skills, a simple realization option should be implemented.

## Conclusions

The subjective effects of a tracheostoma in critically ill patients have not yet been assessed in a standardized way, and instruments for measuring well-being are unavailable. With the development of the TWBS, a valid score is now available, which is easy to apply and allows precise statements about the influence of a tracheostoma on the well-being of critically ill patients. In addition, this score offers potential starting points for the ICU team to increase the well-being of this patient group without having to use additional diagnostic equipment. It is possible that this score will increase the quality of life of concerned patients by improving tracheostomy and weaning management. Further prospective, multicentric research is needed to test the impact of the TWBS among tracheostomized patients.

## Supplementary Information

Below is the link to the electronic supplementary material.

Supplementary file1 (PDF 176 KB)

Supplementary file2 (PDF 109 KB)
