# Supplementary material for: Development of the Tracheostomy Well-Being Score in critically ill patients
Source: Eur J Trauma Emerg Surg. 2022 Oct 13;49(2):981–90. doi: 10.1007/s00068-022-02120-9 (PMC10175326; doi:10.1007/s00068-022-02120-9)
Supplement: Supplementary file 2 — Supplementary file2 (PDF 109 KB) [file 68_2022_2120_MOESM2_ESM.pdf]

Development and validation of the Tracheostomy Well-Being Score in critically ill patients

The European Journal of Trauma and Emergency Surgery

Christopher Ull[1]* (MD), Christina Weckwerth[2]* (MSc), Uwe Hamsen[1] (MD), Oliver Jansen[1] (MD), Aileen Spickermann[1] (MD), Thomas Armin Schildhauer[1] (MD, PhD), Robert Gaschler[2] (PhD), Christian Waydhas[1,3] (MD, PhD)

[1]Department of General and Trauma Surgery, BG University Hospital Bergmannsheil, Bürkle-de-la-Camp-Platz 1, 44789 Bochum, Germany.

[2]Faculty of Psychology, FernUniversität of Hagen, Universitätsstraße 47, 58097 Hagen, Germany.

[3]Medical Faculty University Duisburg-Essen, Hufelandstraße 55, 45147 Essen, Germany.

*Both authors contributed equally as first author.

Corresponding author

Christopher Ull (CU) (ORCID: 0000-0001-5633-3179), Department of General and Trauma Surgery, BG University Hospital Bergmannsheil, Bürkle-de-la-Camp-Platz 1, 44789 Bochum (North Rhine-Westphalia), Germany, e-Mail: christopher.ull@rub.de, phone: +(49)234 302-3576, fax: +(49)234 302-6425.

The 25-items questionnaire of the Tracheostomy Well-Being Score

Respiration:

R1: How often is breathing complicated by your tracheostomy tube?

R2: How often do you experience shortness of breath?

R3: How often do you feel that your trachea is dry?

Coughing:

C1: How often do you cough?

C2: Do you have coughing attacks while speaking?

C3: How often do you feel that you cannot cough up mucus properly?

C4: How often do you need suctioning?

Pain:

P1: How often do you have a foreign body sensation?

P2: How often does your cannula hurt when you swallow?

P3: How often does your cannula hurt when you move or are moved?

P4: How often do you have pain when suctioning?

P5: How often do you have pain when changing your cannula?

Speaking:

S1: How often do you think you are well understood with whispered speech?

S2: How often do you feel the volume of your speech is normal?

S3: How often does your cannula hurt when you speak?

S4: How often do you feel out of breath when speaking?

Swallowing:

SW1: How often does your tracheostomy tube interfere with swallowing liquids or food?

SW2: How often do you feel saliva flowing into the trachea?

SW3: How often are you satisfied with your currently inserted tracheal cannula?

SW4: How often are you afraid of choking or coughing while eating or drinking?

Comfort:

CO1: How often do you feel shame or disgust because of your tracheostomy tube?

CO2: How often are you afraid of your cannula slipping out?

CO3: How often do you feel that your tracheostomy tube fits well?

CO4: How often does an unblocked tracheostomy tube feel more comfortable than a blocked tracheostomy tube?

CO5: How often do you find the fixation of your tracheostomy tube comfortable?

Response options:

4-point Likert scale (0 = never, 1 = sometimes, 2 = often, 3 = always)
